# Comparative Analysis of Tumor Microbiota Identifies a Metastatic-Specific Bacterial Signature, Highlighting *Streptococcus* spp. As the Predominant Hub across Cancers

**DOI:** 10.32604/or.2026.076380

**Published:** 2026-04-22

**Authors:** Nevena Todorovic, Sara Bertorello, Giulia Nannini, Serena Pillozzi, Simonetta Bianchi, Maria Raffaella Ambrosio, Elena Niccolai, Simone Baldi, Amedeo Amedei

**Affiliations:** 1 Department of Experimental and Clinical Medicine, University of Florence, Florence, Italy; 2 Department of Biomedical, Experimental and Clinical Sciences, “Mario Serio” University of Florence, Florence, Italy; 3 Department of Health Sciences, Division of Pathological Anatomy, University of Florence, Florence, Italy; 4 Azienda USL Toscana Nord Ovest, Pathology Unit, Pisa, Italy

**Keywords:** Intratumoral microbiota, lung cancer, breast cancer, colorectal cancer, metastasis, *Streptococcus* spp.

## Abstract

**Background:**

Cancer remains one of the leading global health challenges, with lung cancer (LC), breast cancer (BC), and colorectal cancer (CRC) among the most prevalent and deadly malignancies. The intratumoral microbiota (IM), a distinct microbial ecosystem within tumor tissues, has recently emerged as a potential modulator of carcinogenesis, immune responses, and metastatic progression. However, comparative cross-cancer analyses remain limited. Therefore, this study aimed to compare the IM across these cancer types, with particular emphasis on distinguishing metastatic from non-metastatic malignancies, to identify tumor-specific microbial signatures with potential relevance for biomarker discovery, patient stratification, and microbiota-informed therapeutic strategies.

**Methods:**

We performed 16S rRNA gene sequencing to profile the IM in formalin-fixed, paraffin-embedded (FFPE) samples from 20 BC patients, 20 CRC patients and 15 non-small cell lung cancer (NSCLC) patients.

**Results:**

BC samples exhibited the highest genus-level richness, whereas CRC samples showed significantly greater overall alpha diversity, consistent with the microbial complexity of the gut environment. NSCLC samples displayed the most balanced microbial distribution, as reflected by the highest Shannon index value. Stratification by metastatic status revealed distinct microbial signatures: 16 genera were exclusive to metastatic tumors and 49 to non-metastatic ones. In BC specifically, the class Clostridia and the family Burkholderiaceae were enriched in non-metastatic samples, accompanied by functional shifts in pantothenate and coenzyme A biosynthesis, lysine metabolism, and lipid A pathways. Microbial network analysis further revealed differences in ecological community structure and keystone taxa: *Streptococcus* spp. predominated as hubs in metastatic tumors, whereas *Neisseria* spp. were central in non-metastatic networks.

**Conclusions:**

Overall, our findings highlight cancer-type and metastasis-specific microbial signatures, supporting a potential role for the IM in tumor progression and offering novel avenues for biomarker discovery and therapeutic targeting.

## 1 Introduction

Cancer remains one of the greatest global health challenges, demanding integrated, multidisciplinary research efforts. According to GLOBOCAN 2022, lung cancer (LC) was the leading cause of cancer-related mortality, accounting for 1.8 million deaths (18% of all cancer fatalities) [1]. In 2022, breast cancer (BC) was diagnosed in 2.3 million women and caused approximately 670.000 deaths, while colorectal cancer (CRC) ranked third in incidence (10% of new cases) and second in mortality, with over 1.9 million new diagnoses and 930.000 deaths worldwide [2]. The heavy burden of LC, BC, and CRC thus motivated our focus on these three major public health threats. Over the past decade, the host microbiome has emerged as a key modulator of cancer onset, progression, diagnosis, and treatment. It influences tumor biology primarily through three mechanisms: (i) altering the balance between cell proliferation and apoptosis, (ii) modulating immune responses, and (iii) affecting the metabolism of endogenous compounds and therapeutic agents [3]. Advances in sequencing and computational approaches have identified the intratumoral microbiota (IM) as an integral component of the tumor microenvironment. Specifically, the IM can promote genomic instability, drive epigenetic alterations, sustain pro-tumor inflammation, enable immune evasion, reprogram cellular metabolism, and ultimately support invasion and metastasis [4]. Anatomical and physiological distinctions among organs, such as the densely colonized gut, the immune-modulatory breast tissue, and the barrier-protected lung, likely shape IM composition and function in cancer-specific ways. However, although numerous studies have profiled the IM within specific tumor types, cross-cancer comparative analyses remain scarce. As a result, it is unclear whether observed microbial differences reflect tumor specificity, tissue-driven factors, or broader host–microbiota interactions. In this context, the IM is increasingly recognized as a potential diagnostic and prognostic biomarker, supported by studies reporting microbial profiles associated with specific tumor types and subtypes [5,6]. Moreover, growing evidence suggests a potential therapeutic role for the IM in modulating cancer progression and treatment response [7–9]. Nonetheless, current findings are sometimes contradictory, and both diagnostic and functional applications of the IM remain under active debate. In light of the IM’s emerging importance in the tumor microenvironment and its potential as a source of clinically relevant biomarkers, the present study provides a comprehensive cross-cancer characterization of the IM in three high-burden malignancies (LC, BC, CRC) using formalin-fixed, paraffin-embedded (FFPE) tumor samples from an Italian cohort. Specifically, we aimed to (i) profile and compare IM diversity, taxonomic composition, and predicted functional features across these tumor types, and (ii) determine whether distinct microbial signatures are associated with metastatic vs. non-metastatic status within each cancer. By integrating comparative and stratified analyses, this work seeks to clarify whether IM patterns reflect tissue- and tumor-specific microbial ecosystems and to identify microbial traits with potential diagnostic, prognostic, and therapeutic relevance.

## 2 Materials and Methods

### 2.1 Samples Collection

FFPE tissue samples were obtained from patients with BC, CRC, and non-small cell lung cancer (NSCLC) at the Careggi University Hospital, Florence, Italy. The study cohort included 20 BC patients (10 males and 10 females; mean age 60.35 ± 17.22 years; Table S1), 20 CRC patients (7 males and 13 females; mean age 62.25 ± 14.14 years; Table S2), and 15 NSCLC patients (9 males and 6 females; mean age 77.00 ± 17.22 years; Table S3). FFPE samples from both male and female patients for each cancer type were included to mirror the real-world composition of the disease and to avoid sex-based selection bias, as the study aimed to analyze tumor-related features rather than sex-specific differences. The study was conducted in accordance with the Declaration of Helsinki and approved by Comitato Etico Regione Toscana—Area Vasta Centro (CEAVC) (No. 19542). The requirement for written informed consent was waived due to the retrospective nature of the study.

To minimize environmental contamination, the first microtome scrolls from each FFPE block were discarded. Subsequently, 8–10 sections (thickness up to 10 µm; surface area up to 250 mm²) were cut and collected into sterile 2-mL centrifuge tubes. Paired blank paraffin samples from the same FFPE blocks were collected in parallel as negative controls. The microtome was thoroughly cleaned between each sample, and routine contamination checks were performed on all equipment to ensure sample integrity.

### 2.2 IM Characterization

Genomic DNA was extracted from FFPE sections using the QIAamp DNA FFPE Advanced Kit (cat. No. 56604, Qiagen, Hilden, Germany), according to the manufacturer’s instructions. DNA concentration and purity were assessed by NanoDrop ND-1000 (Thermo Fisher Scientific, Waltham, MA, USA) and Qubit Fluorometer (Thermo Fisher Scientific, Waltham, MA, USA), and then it was stored at-20°C. Next, extracted DNA samples were sent to IGA Technology Services (I-33100 Udine, Italy), where amplicons of the V3-V4 hypervariable region of the bacterial 16S rRNA gene were sequenced in paired-end (2 × 300 pb) on the Illumina Miseq platform.

Raw paired-end FASTQ reads were processed using the MICCA (MICrobial Community Analysis) software pipeline [10]. Demultiplexed sequence reads were processed using QIIME2 (v.2021.4). The Cutadapt tool was employed to discard sequences lacking primers while DADA2 was used to trim low-quality nucleotides from both forward and reverse reads and to perform paired-end filtering, merge reads and remove chimeras. Host-derived sequences were identified by aligning amplicon sequence variants (ASV) to the GRCh38 human reference genome with Bowtie2 (v.2.4.4) and subsequently discarded. The remaining ASVs were imported into QIIME2, and taxonomic classification was performed through the Scikit-learn multinomial Naive Bayes classifier re-trained on the Silva database (release 138) for the V3–V4 region. To minimize noise, ASVs with mean relative abundance below 0.005% were excluded [11], and reads assigned to Chloroplast and Mitochondria were removed [12].

### 2.3 Statistical Analyses

Statistical analyses of bacterial communities were performed in R (v.4.2.2) using the following packages: phyloseq (v.1.42.0), vegan (v.2.6-4), DEseq2 (v.1.38.3), ggplot2 (v.3.2.4), and other packages satisfying their dependencies. Saturation analysis was conducted for each sample using the rarecurve function. The observed richness and Shannon indices were used to perform alpha diversity analyses. Pielou’s evenness index was calculated as E = S/log (R), where S is the Shannon index and R is the observed ASV richness. Group differences were tested using the Wilcoxon or Kruskal-Wallis tests, with *p*-values < 0.05 considered statistically significant. For significant results, Dunn’s post hoc test was applied for pairwise comparisons. Permutational ANOVA (PERMANOVA) with 9999 permutations was applied to beta diversity distance matrices (calculated using Hellinger distance on Hellinger-transformed genera abundances) to test the significance of sample clusters observed following principal coordinate analysis (PCoA). Differential abundance (DA) analysis was performed at all taxonomic ranks using the DESeq2 algorithm on raw count data. Significant DA results were identified based on an adjusted *p*-value < 0.05 after Benjamini–Hochberg correction for multiple testing. To reduce noise, only taxa with a grand mean count ≥100 were retained in the displayed results. Genus-level Venn diagrams were generated using the ggvenn (v.0.1.10) package. Predicted MetaCyc pathway abundances were inferred through PICRUST2 (v2.4.2) with the EPA-ng algorithm, and significant differences among groups were identified with LEFSE (LDA Effect Size) analysis. Finally, to explore group-specific microbial co-occurrence, network analysis of the top 20 most abundant genera per group was performed using the NetCoMi package (v.1.1.0).

## 3 Results

### 3.1 IM Composition across Cancer Types

The IM composition of the three tumor types was characterized by five differently predominant bacterial phyla. In detail, for BC samples, the most abundant phyla were Proteobacteria (42.6%), Firmicutes (31.1%), Actinobacteriota (15.1%), Bacteroidota (7.5%), and Fusobacteriota (1.1%) (Fig. S1A). CRC samples were dominated by Firmicutes (51.9%), followed by Bacteroidota (18.8%), Proteobacteria (13.6%), Actinobacteriota (11.0%), and Fusobacteriota (2.7%) (Fig. S1B). Finally, in NSCLC samples, the most abundant phyla were Firmicutes (39.1%), Bacteroidota (38.3%), Proteobacteria (4.9%), Campylobacterota (5.0%), and Actinobacteriota (4.4%) (Fig. S1C).

At the genus-level, BC tissues were enriched in *Sphingomonas* (17.4%), *Streptococcus* (10.7%), *Acinetobacter* (4.9%), *Cutibacterium* (4.8%) and *Corynebacterium* (4.0%) (Fig. S1D). In CRC, the most abundant genera were *Streptococcus* (22.9%), *Prevotella* (7.7%), *Bacteroides* (6.2%), *Neisseria* (5.1%), and *Veillonella* (4.4%) (Fig. S1E) while NSCLC samples exhibited a distinct profile dominated by *Muribaculaceae* (18.2%), *Lactobacillus* (9.2%), *Prevotellaceae UCG-001* (5.2%), *Helicobacter* (4.7%), and *Streptococcus* (4.6%) (Fig. S1F).

To compare bacterial genus richness among the three cancer cohorts, we generated a histogram of observed genera following quality filtering. BC samples displayed significantly greater genus richness than both NSCLC and CRC specimens (Fig. S2).

**Figure 1:**
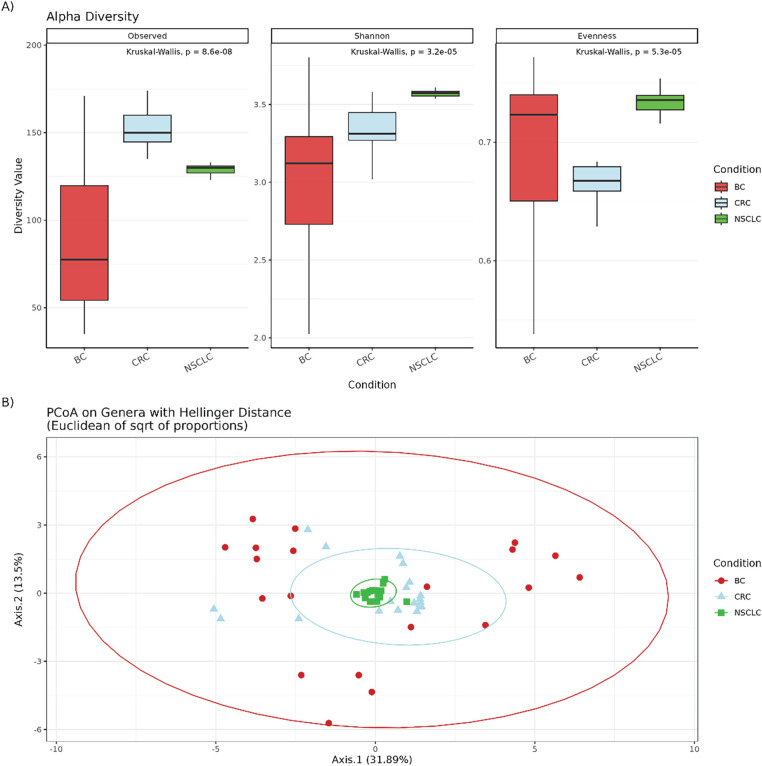
(**A**) Box plots representing alpha diversity indices (Observed ASV richness, Shannon index, and Pielou’s Evenness) across BC, CRC, and NSCLC samples. Statistical differences were assessed using the Kruskal-Wallis test and *p*-values < 0.05 were considered significant. Pairwise differences between groups were further analyzed using Dunn’s post hoc test. (**B**) Principal Coordinate Analysis (PCoA) based on Hellinger distances of transformed genus-level abundances. Group differences were assessed using Permutational Multivariate Analysis of Variance (PERMANOVA) with 9999 permutations applied to the beta diversity distance matrices. BC: Breast cancer; CRC: Colorectal cancer; NSCLC: Non-small cell lung cancer.

All three alpha-diversity metrics, observed richness, Shannon index and Pielou’s evenness, differed significantly among cancer types (Fig. 1A). CRC samples exhibited significantly higher richness compared to both BC (adj. *p* < 0.0001) and NSCLC (adj. *p* = 0.001) samples. In contrast, NSCLC tissues showed a significant higher Shannon index compared to BC (adj. *p* < 0.0001) and CRC (adj. *p* = 0.002) ones. In addition, CRC samples displayed significantly lower evenness compared to NSCLC (adj. *p* < 0.0001) and BC (adj. *p* = 0.02), whereas NSCLC samples showed greater evenness than BC (adj. *p* = 0.02). Conversely, by PCoA of Hellinger-transformed genus abundances, intergroup differences in IM community structure were not significant (*p* = 0.9) (Fig. 1B).

### 3.2 Shared IM Bacteria

**Figure 2:**
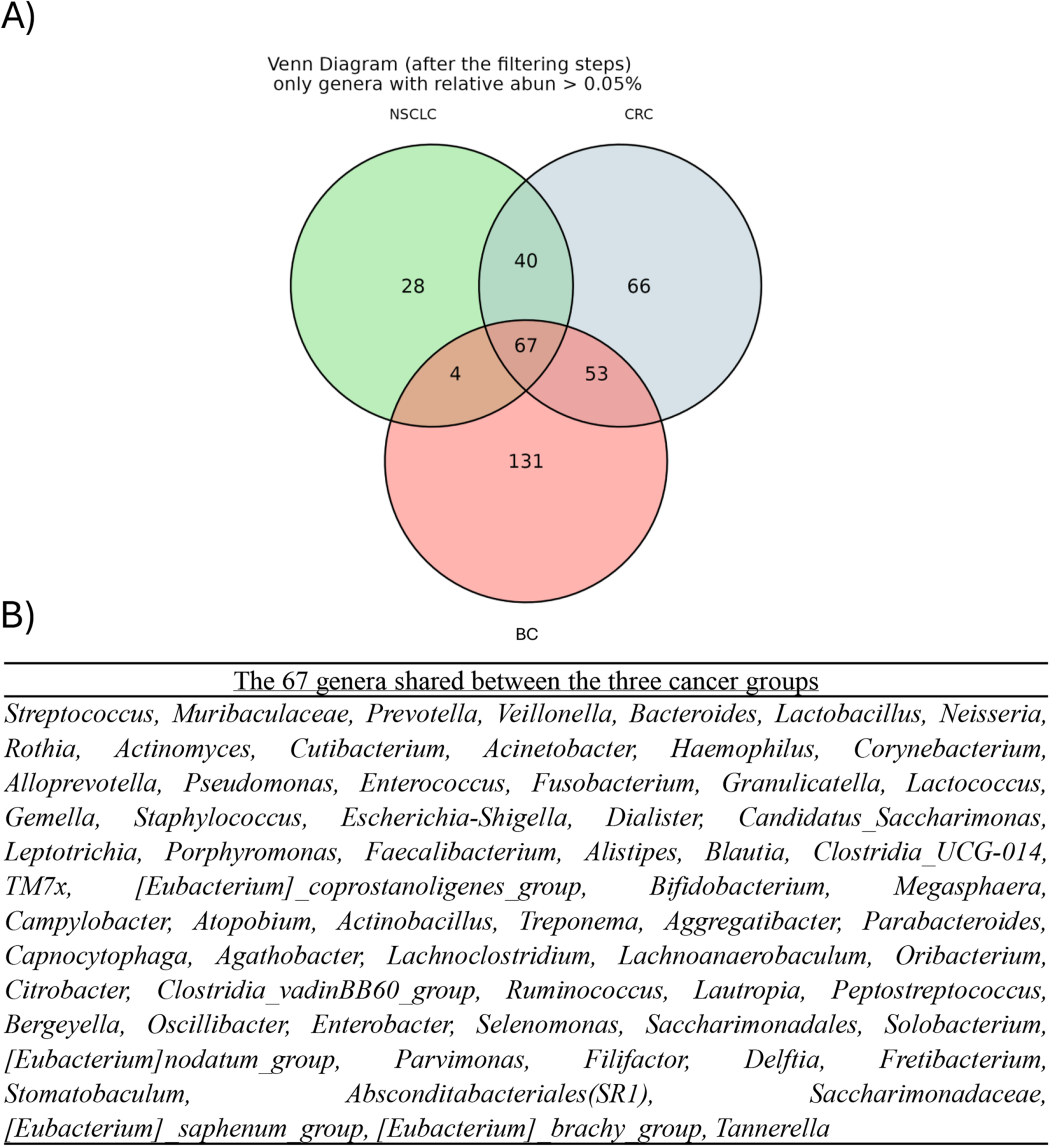
(**A**) Venn diagram showing shared and unique genera among BC, CRC, and NSCLC samples. (**B**) Shared genera between the three tumor types, ranked by relative abundance. BC: Breast cancer; CRC: Colorectal cancer; NSCLC: Non-small cell lung cancer.

To delineate tumor-associated microbial signatures, we first compared the presence and exclusivity of bacterial genera across CRC, BC and NSCLC samples. A Venn analysis (Fig. 2A) identified 67 genera common to all three tumor types; in contrast, 28 genera were unique to NSCLC, 66 to CRC and 131 to BC. We then ranked these shared and IM-specific genera by their mean relative abundances within each cancer cohort (Fig. 2B). This ranking highlights the key microbial players that are both pervasive across multiple tumor niches and those that may contribute to cancer-type–specific IM ecosystems.

### 3.3 Differences in IM among Metastatic and Non-Metastatic Cancers

**Figure 3:**
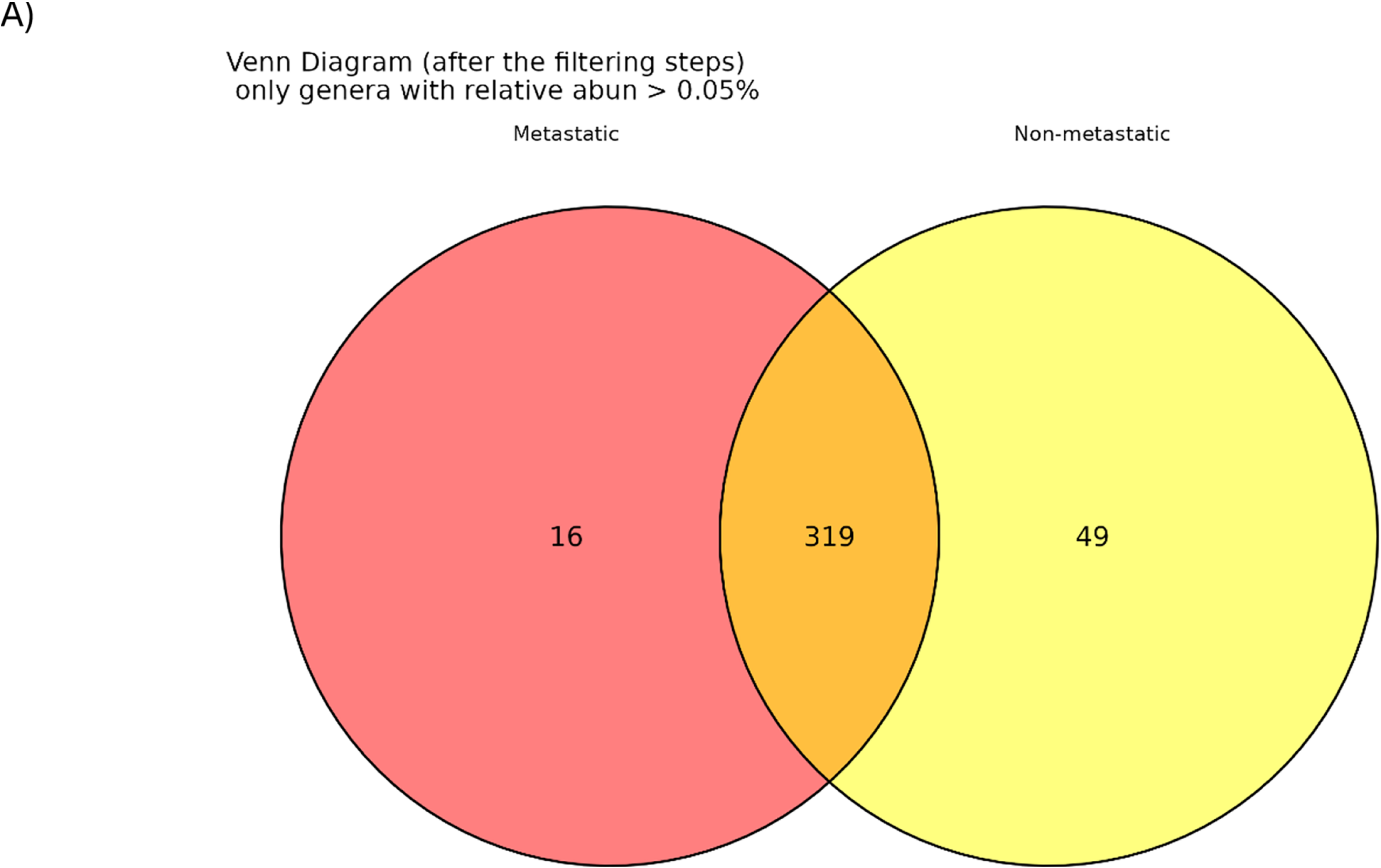

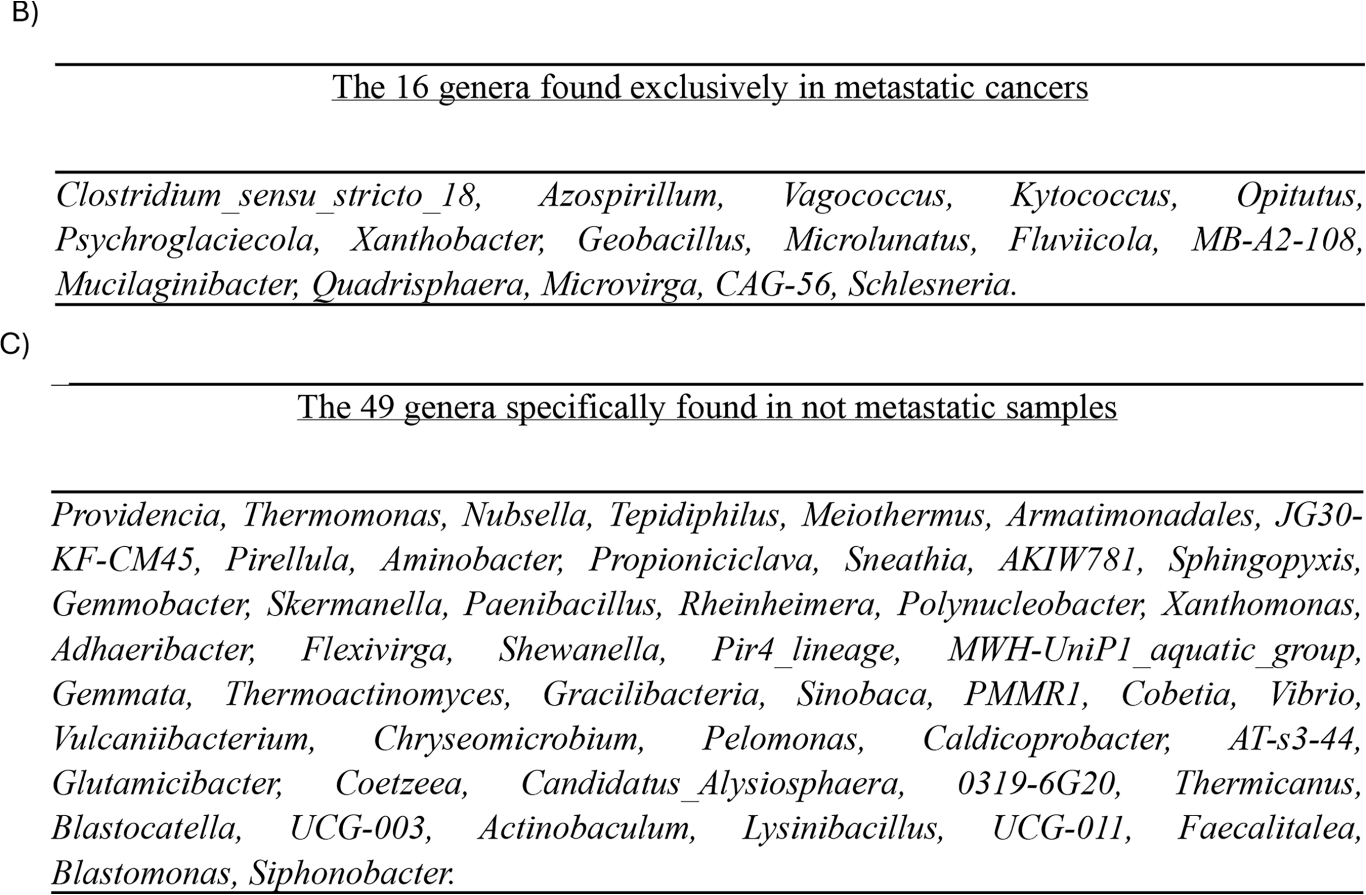
(**A**) Venn diagram illustrating the comparison of bacterial genera identified in the intratumoral microbiota of metastatic and non-metastatic cancer samples. (**B**) Bacterial genera found exclusively in metastatic cancer patients, ranked in descending order based on their relative abundance. (**C**) Bacterial genera identified only in non-metastatic cancer patients, ranked by relative abundance.

We next stratified tumors into metastatic and non-metastatic groups (Fig. 3A): BC included 12 non-metastatic and 8 metastatic cases, CRC included 13 non-metastatic and 7 metastatic cases, and NSCLC included 8 non-metastatic and 7 metastatic cases. Across all cancer types combined, 319 genera were shared between metastatic and non-metastatic samples, while 16 genera were unique to metastatic tumors (Fig. 3B) and 49 genera were exclusive to non-metastatic tumors (Fig. 3C). Notably, none of these subgroup-specific genera were present across all three cancer types, suggesting that cancer-intrinsic factors shape IM differences between metastatic and non-metastatic settings.

**Figure 4:**
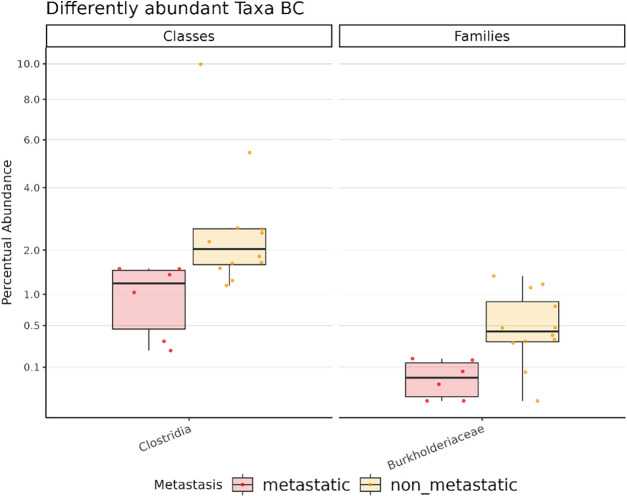
Box plot showing the significant differentially abundant taxa among metastatic and non-metastatic BC samples. All results have an adjusted. *p* < 0.05. BC: Breast cancer.

To investigate whether metastasis is associated with shared microbial patterns across tumor types, we first performed a combined analysis of all cancers, comparing metastatic and non-metastatic samples. However, no significant differences in either alpha (Fig. S3A) or beta (Fig. S3B) diversity between the two groups were observed. To assess potential cancer-type-specific effects, we then conducted separate alpha (Fig. S4A) and beta (Fig. S4B) diversity analyses within each cancer type but inter-group comparisons did not show statistically significant differences. Next, to identify microbial taxa associated with metastasis independently of tumor origin, we performed a global differential abundance analysis across all cancer types; however, no statistically significant differences were found (data not shown). Notably, DESeq2 algorithm revealed significant taxonomic shifts within BC samples; specifically, the class Clostridia (adj. *p* = 0.003) and the family Burkholderiaceae (adj. *p* < 0.0001) were significantly enriched in non-metastatic samples compared to metastatic ones. (Fig. 4).

**Figure 5:**
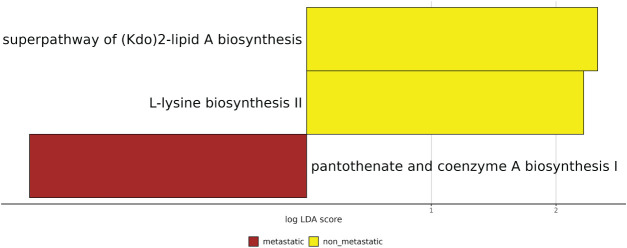
Statistically significant different predicted pathways with LDA score >3.0 between metastatic and non-metastatic patients. All results have a *p* < 0.05. LDA: Linear discriminant analysis.

Finally, we compared predicted functional profiles between metastatic and non-metastatic tumors. An initial global analysis across all cancer’s types revealed no significant pathway differences (data not shown), so we conducted pairwise comparisons within each cancer cohort. Of note, significant functional alterations were detected only in the BC cohort; in particular, the pantothenate and coenzyme A biosynthesis I pathway was enriched in metastatic tumors (LDA score = 2.09, *p* = 0.01) whereas the L-lysine biosynthesis II pathway (LDA = 2.22, *p* = 0.04) and the superpathway of (Kdo)_2_-lipid A biosynthesis (LDA = 2.33, *p* = 0.05), were enriched in non-metastatic BC samples (Fig. 5).

### 3.4 Microbial Interaction Networks: Metastatic vs. Non-Metastatic Tumors

**Figure 6:**
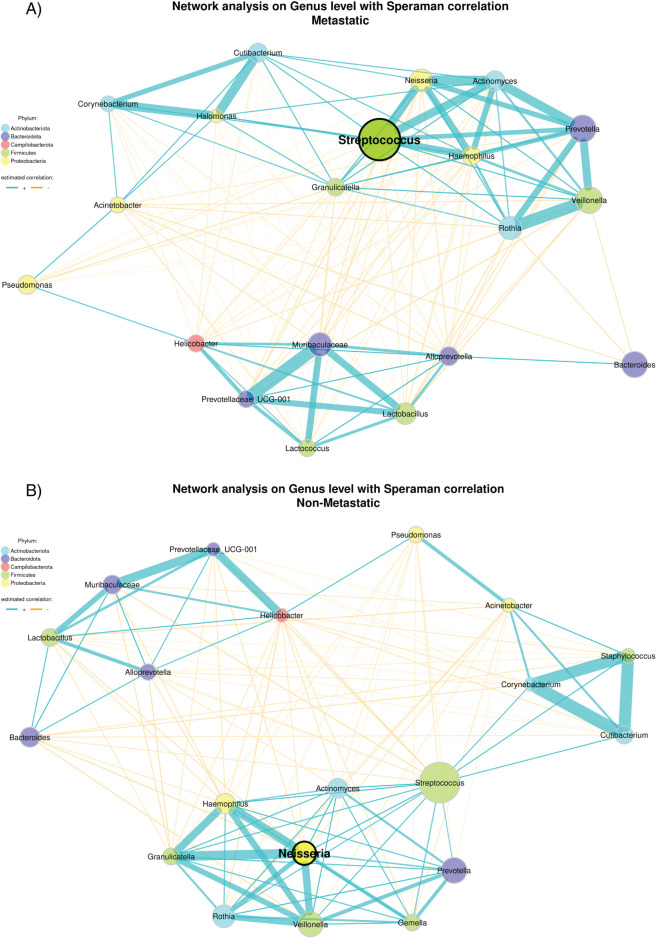
Interaction networks of the top 20 bacterial genera in the metastatic (**A**) and non-metastatic (**B**) groups. Nodes represent bacterial genera and are colored according to their respective phylum (see legend). Node size reflects the number of associations (degree centrality), with larger nodes indicating taxa with more interactions. The hub taxon, defined as the genus with the highest eigenvector centrality, is outlined in black. Edges represent significant associations between taxa, with blue lines indicating positive correlations and orange lines indicating negative correlations. The thickness of the edges corresponds to the strength of the correlation. The networks were constructed using centered log-ratio (CLR) normalized abundance data and visualized using a spring layout.

Microbial co-occurrence networks were constructed separately for metastatic and non-metastatic tumors using the 20 most abundant genera after centered log-ratio (CLR) normalization. In the metastatic network, the average shortest path length was 1.27, edge density 0.73, and edge connectivity 4; *Streptococcus* spp. emerged as the genus with the highest eigenvector centrality (Fig. 6A). By contrast, the non-metastatic network displayed an average path length of 1.36, edge density of 0.64, and edge connectivity of 10, with *Neisseria* spp. as the most central taxon by eigenvector centrality (Fig. 6B).

To explore tumor-type–specific community structures, we next constructed microbial co-occurrence networks for metastatic and non-metastatic BC subgroups. In the metastatic BC group, the network exhibited an average path length of 1.37, edge density of 0.65, and edge connectivity of 2, documenting a moderately cohesive structure with limited robustness to disconnection. The network consisted of a single connected component. *Anaerobacillus* spp. emerged as the primary hub, with the highest eigenvector centrality (Fig. S5A). In contrast, the non-metastatic BC network exhibited a shorter average path length (1.24), higher edge density (0.76), and greater edge connectivity, reflecting a more tightly interconnected and resilient community; of note, *Granulicatella* spp. was identified as the central hub (Fig. S5B).

For CRC, we similarly constructed microbial co-occurrence networks stratified by metastatic status. The metastatic CRC network was highly compact and displayed several microbial interconnections: it exhibited an average shortest-path length of 1.07, an edge density of 0.93, and an edge connectivity of 11, all within a single connected component. *Actinomyces* emerged as the principal hub genus, possessing the highest eigenvector centrality (Fig. S6A). In contrast, the non-metastatic CRC network was looser and less cohesive, with a longer average path length (1.46), lower edge density (0.54), and edge connectivity of 4. Despite being less densely connected, the network also formed a single connected component, with *Enterococcus* serving as the central hub genus. (Fig. S6B).

Finally, the application of network analysis to NSCLC samples revealed largely conserved topologies between metastatic and non-metastatic groups. The metastatic group showed an average path length of 1.48, an edge density of 0.53, and edge connectivity of 4, indicating a moderately connected network with a single cohesive component. The genus *Streptococcus* emerged as the central hub, displaying the highest eigenvector centrality (Fig. S7A). Likewise, the non-metastatic group showed a similar average path length (1.45), a slightly higher edge density (0.55), and the same edge connectivity [4], again with *Streptococcus* spp. at its center (Fig. S7B).

## 4 Discussion

In this study we compared IM composition and inferred function across different cancer types using FFPE samples, with a particular focus on microbial interaction networks in metastatic vs. non-metastatic tumors. Specifically, we profiled microbial composition and diversity in BC, CRC and NSCLC, yielding several key insights.

In an initial overview of community composition, BC samples exhibited the highest genus richness, consistent with previous reports identifying BC as relatively microbially rich among tumor types [13,14]. However, alpha diversity analysis revealed significantly greater ASV richness in CRC compared with both BC and NSCLC, suggesting a more complex cancer-associated microbial ecosystem in CRC. This finding aligns with the gastrointestinal tract’s unique anatomy, physiology, and environmental exposures and with extensive literature linking the gut microbiome to CRC pathogenesis [15–17]. Other alpha-diversity indices also varied by cancer type: NSCLC samples displayed the highest Shannon index, reflecting a more even genus distribution. Collectively, these results support the notion that each cancer harbors a distinct IM signature, shaped by tissue-specific features and the tumor microenvironment. Nevertheless, despite these alpha-diversity differences, beta-diversity analyses did not reveal clear compositional separation across the three cancer types.

All five predominant phyla, Proteobacteria, Firmicutes, Actinobacteriota, Bacteroidota, and Fusobacteriota, were detected in each cancer type, albeit with cancer-specific relative abundances. At the genus level, BC samples were enriched in *Sphingomonas*, *Streptococcus*, *Acinetobacter*, *Cutibacterium*, and *Corynebacterium*; CRC samples were dominated by *Streptococcus*, *Prevotella*, *Bacteroides*, *Neisseria*, and *Veillonella*; and NSCLC samples were enriched in *Muribaculaceae*, *Lactobacillus*, *Prevotellaceae_UCG-001*, *Helicobacter*, and *Streptococcus*.

Across cancers, 67 genera were shared, while BC harbored 131 unique genera, CRC 66, and NSCLC 28. These observations highlight the influence of tissue architecture, anatomical location, and immune context in shaping IM communities [18]. The relatively low number of genera in NSCLC may reflect the lung’s continuous exposure to the external environment and its efficient clearance mechanisms, which together maintain a tightly regulated microbiome even under disease conditions [19]. Intriguingly, CRC shared more genera with both BC and NSCLC than BC and NSCLC shared with each other, despite closer anatomical proximity between BC and NSCL, suggesting a potential systemic influence of gut microbiome on distant tumors [20].

We next stratified samples by metastatic status to investigate IM alterations associated with metastatic progression. Among the 319 genera shared across all samples, 16 were unique to metastatic tumors and 49 were unique to non-metastatic tumors, suggesting that metastatic progression may exert selective pressure on the IM and drive distinct community-level changes [21]. However, none of the 16 metastatic-specific genera were shared across all cancer types, indicating that metastasis-related IM shifts are tumor-specific rather than universal. Consistent with this interpretation, global and within-cancer comparisons (BC, CRC, NSCLC) revealed no significant differences in alpha or beta diversity between metastatic and non-metastatic groups. Differential-abundance analysis identified signals only in BC: two taxa, the class Clostridia and the family Burkholderiaceae, were enriched in non-metastatic tumors. This contrasts with earlier reports linking Burkholderiaceae to aggressive BC subtypes and with observations of elevated Burkholderiaceae in BC tissue vs. healthy controls [22,23]. Such discrepancies may reflect genus- or strain-level heterogeneity within the Burkholderiaceae family, with different members exerting divergent effects on tumor biology.

Interestingly, when all metastatic samples were compared globally to all non-metastatic samples irrespective of cancer type, no significant compositional differences were observed. This further supports the concept that metastasis-related IM alterations are largely tumor-type specific, consistent with recent findings by Kanimdan and colleagues in FFPE samples from the same cancer types [24].

Furthermore, to examine whether metastatic progression is accompanied by functional IM shifts, we applied PICRUSt2 to infer metagenomic pathways. No pathways reached statistical significance in the metastatic vs. non-metastatic comparison, indicating that aggregate microbial functional potential remains largely unchanged with metastatic status. Tumor-type–stratified analyses identified significant differences only in BC: the pantothenate and coenzyme A biosynthesis I pathway was enriched in metastatic tumors, potentially supporting bacterial fatty acid metabolism and energy generation [25], whereas L-lysine biosynthesis II and the superpathway of (Kdo)_2_-lipid A biosynthesis were enriched in non-metastatic BC. Enrichment of lipid A biosynthesis in non-metastatic tumors may reflect differences in Gram-negative taxa that promote an immune microenvironment more conducive to immunosurveillance than that observed during metastatic progression [26,27].

Finally, to identify potential keystone taxa, we constructed co-occurrence networks both globally and within each cancer type, comparing metastatic and non-metastatic tumors.

Although overall network architecture was broadly similar, a notable hub transition was observed: *Streptococcus* spp. emerged as the dominant hub in metastatic tumors, whereas *Neisseria* spp. served as the hub in non-metastatic tumors.

The role of *Streptococcus* spp. in cancer appears highly context dependent. In BC, enrichment of *Streptococcus* in metastatic tumors is consistent with previous reports showing that specific species promote dissemination by inhibiting RhoA–ROCK signaling and inducing cytoskeletal remodeling [28]. By contrast, in CRC, *Streptococcus* may exert protective rather than tumor-promoting effects. In a genus-level study by Li et al., co-culture with *Streptococcus thermophilus* or its conditioned medium decreased CRC cell proliferation, while oral gavage with this bacterium significantly suppressed tumorigenesis *in vivo*. Moreover, patients whose tumors were enriched in *Streptococcus* spp. showed better clinical outcomes compared with those harboring lower levels of these bacteria [29]. Mechanistically, β-galactosidase secreted by *S. thermophilus* was documented to inhibit CRC cell proliferation, diminish colony formation, induce cell-cycle arrest, and promote apoptosis, thereby suppressing tumor growth [30]. Nevertheless, *Streptococcus* has also been found to be one of the ten predominant genera in metastatic CRC [31].

*Neisseria* spp. also exhibit context-dependent behavior: pathogenic species such as *N. gonorrhoeae* can promote carcinogenesis through chronic inflammation [32], whereas commensal species such as *N. sicca* contribute to mucosal homeostasis and genomic stability [33]. In line with this dual role, unclassified Neisseriaceae have been inversely correlated with PD-L1 expression in lung tumors, suggesting a more favorable immune microenvironment when these taxa are present [34].

When stratified by cancer type, BC exhibited the most pronounced network reorganization between metastatic and non-metastatic tumors. *Granulicatella* spp. acted as a central hub in non-metastatic tumors but lost connectivity in metastatic tumors, where *Anaerobacillus* spp. emerged as a new hub, indicative of IM restructuring during metastatic progression [35]. Interestingly, *Granulicatella* spp. were enriched in the saliva of non-metastatic BC patients yet elevated in feces from patients with high-grade BC, an apparently contradictory pattern that underscores the context-specific behavior of bacteria across body sites and microbial niches [36,37]. Conversely, *Anaerobacillus* spp., previously linked to NSCLC recurrence risk, may represent a metastasis-associated keystone taxon in BC [38].

In CRC, metastatic networks were centered on *Actinomyces* spp., while non-metastatic networks were dominated by *Enterococcus* spp. *Actinomyces* spp. may promote inflammation via TLR2/4–NF-κB signaling and extracellular-vesicle–mediated mitochondrial damage [39–41], whereas *Enterococcus faecalis* plays dual roles in CRC, exerting both pathogenic and probiotic effects depending on context [42]. Notably, *Fusobacterium nucleatum*, commonly implicated in CRC, was absent from non-metastatic networks despite prior associations with lymph node metastasis [43].

In NSCLC, *Streptococcus* spp. remained the dominant hub in both metastatic and non-metastatic networks, suggesting a more stable IM architecture in lung tumors irrespective of metastatic status [44].

Although largely descriptive, these analyses align with a growing body of evidence suggesting that intratumoral microbes may serve not only as biomarkers but also as potential therapeutic targets or modulators [45,46]. Emerging studies demonstrate that specific bacterial taxa can contribute to tumor progression by inducing DNA damage, modulating inflammatory or oncogenic pathways, or shaping an immunosuppressive microenvironment [47–49]. Conversely, other microbial components may enhance antitumor immunity by activating innate immune pathways, promoting antigen presentation, or supporting T- and NK-cell responses.

These findings have stimulated interest in therapeutic strategies aimed at manipulating tumor-resident microbial communities, including selective antimicrobials, bacteriophage therapy, engineered bacteria delivering immunomodulatory molecules, and microbiota-derived adjuvants [50,51].

Within this context, our observation that *Streptococcus* spp. emerge as a central microbial hub in metastatic tumors raises the hypothesis that targeting species within this genus, or the microbial interactions they orchestrate, may offer a novel strategy to modulate metastatic behavior. Although these possibilities remain speculative and require mechanistic validation, they highlight the translational potential of integrating microbial profiling into therapeutic development pipelines.

Despite the novel insights offered by this pilot study, some limitations must be acknowledged. First, the relatively small sample size reduces statistical power to detect subtle IM differences. Second, potential confounders, such as sex, diet, medication history, and tumor stage were not fully controlled and may have influenced microbial profiles. Third, while 16S rRNA sequencing provides broad taxonomic resolution, it cannot resolve strain-level variation or fully characterize functional capacity. Indeed, our prior work showed that IM modulates local immunity in HPV-related CRC: HPV-positive tumors exhibited reduced immune surveillance but enhanced T-cell cytotoxicity compared with HPV-negative cases, alongside distinct microbial profiles [17]. Similarly, we previously reported that in CRC, *Prevotella* spp. abundance negatively correlates with IL-17A but positively correlates with IL-9, whereas *Bacteroides* spp. show an inverse correlation with IL-9, underscoring the need for functional or metabolomic follow-up [52]. Together, these findings highlight the importance of complementary metagenomic or metabolomic analyses using *ex vivo* or frozen cancer tissues to validate biological relevance. Fourth, the cross-sectional design precludes causal inference or assessment of temporal dynamics in cancer progression; longitudinal studies are therefore required to determine whether observed microbial shifts precede or follow metastasis. Fifth, reliance on FFPE tissues, although enabling the use of archival specimens, may introduce biases due to DNA degradation and differential recovery. Nonetheless, recent work in BC has shown that FFPE samples yield microbiome profiles comparable to those obtained from fresh-frozen tissues [53]. Finally, the low microbial biomass typical of tumor samples presents inherent challenges related to contamination risk and analytical noise, underscoring the necessity for rigorous controls and external validation using complementary approaches.

## 5 Conclusion

This study provides a comprehensive overview of the IM across three distinct tumor types: BC, NSCLC and CRC. We documented that each tumor type harbors a unique IM shaped by its tissue architecture and microenvironment. Specifically, breast tumors exhibited the highest genus richness, colorectal tumors displayed the greatest overall diversity, and lung tumors showed the most even genus distribution. Despite these differences, all tumor types shared a core IM composed of five predominant phyla, suggesting the presence of a baseline microbial community that is subsequently modulated in a cancer-specific manner. Beyond this shared foundation, each tumor type also contained numerous unique genera aligned with its anatomical and physiological contexts, reinforcing the specificity of microbial colonization.

Importantly, although we observed clear IM differences between metastatic and non-metastatic lesions and identified *Streptococcus* spp. as a predominant hub genus in metastatic samples, these alterations did not converge into a universal, cross-cancer “metastatic signature.”

Instead, metastasis-associated microbial shifts remained strongly tumor-type specific, emphasizing that microbial dynamics are tightly coupled to the unique biological context of each cancer.

Collectively, these findings underscore that the IM represents an integral and tumor-specific component of the cancer microenvironment and support the notion that deciphering microbial contributions to tumor progression may offer new opportunities for biomarker discovery, patient stratification, and ultimately, microbiota-informed therapeutic interventions.

## Supplementary Materials



## Data Availability

The data presented in this study are deposited in the NCBI Gene Expression Omnibus (GEO) repository, accession numbers: GSE304021, GSE212891 and GSE216589.

## Supplementary Materials

The supplementary material is available online at https://www.techscience.com/doi/10.32604/or.2026.076380/s1.
