# Supplementary material for: Comparative Analysis of Tumor Microbiota Identifies a Metastatic-Specific Bacterial Signature, Highlighting Streptococcus spp. As the Predominant Hub across Cancers
Source: Oncol Res. 2026 Apr 22;34(5):15. doi: 10.32604/or.2026.076380 (PMC13126416; doi:10.32604/or.2026.076380)
Supplement: Supplementary file 1 [file OncolRes-34-76380-s001.docx]

**SUPPLEMENTARY FIGURES**


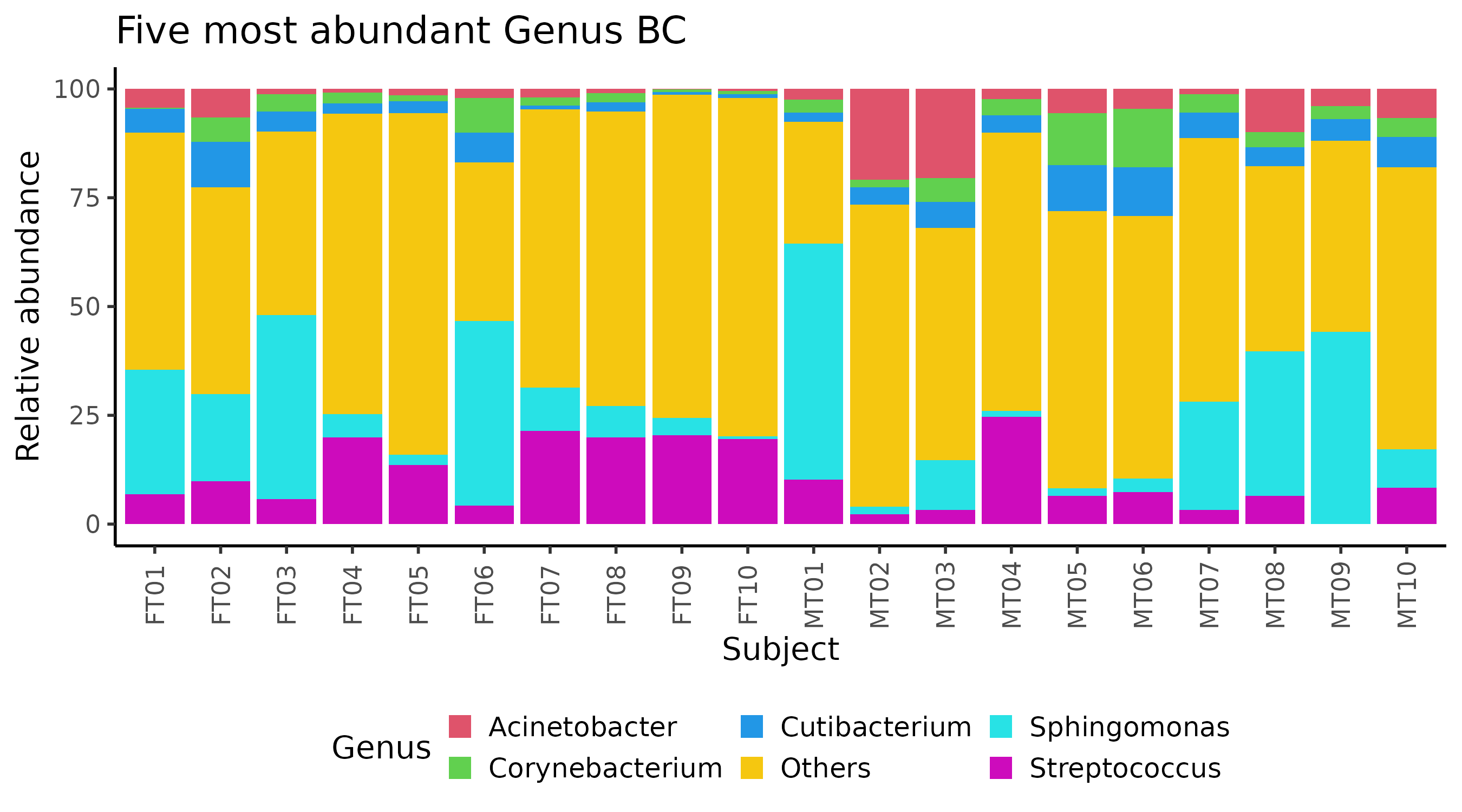


A)


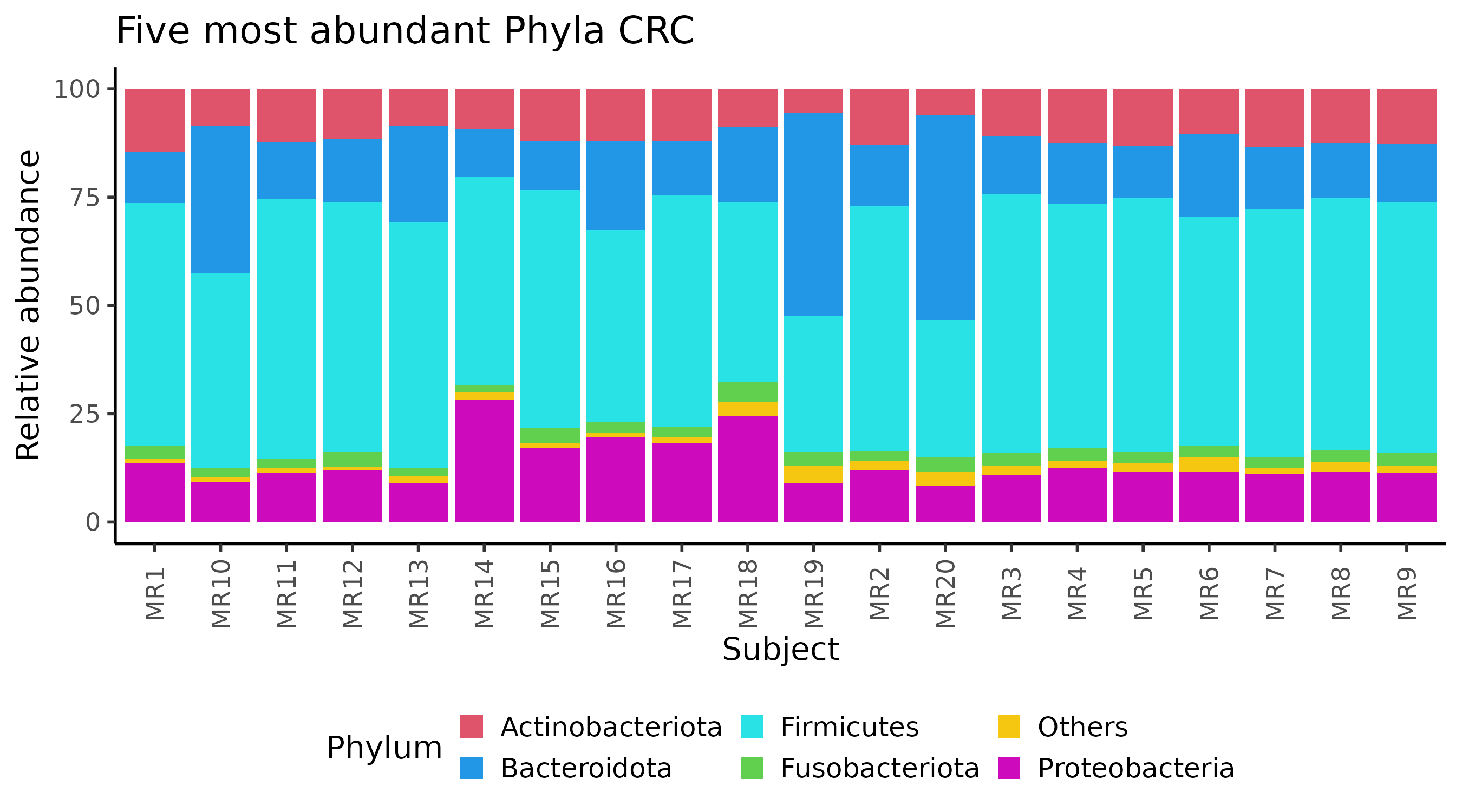

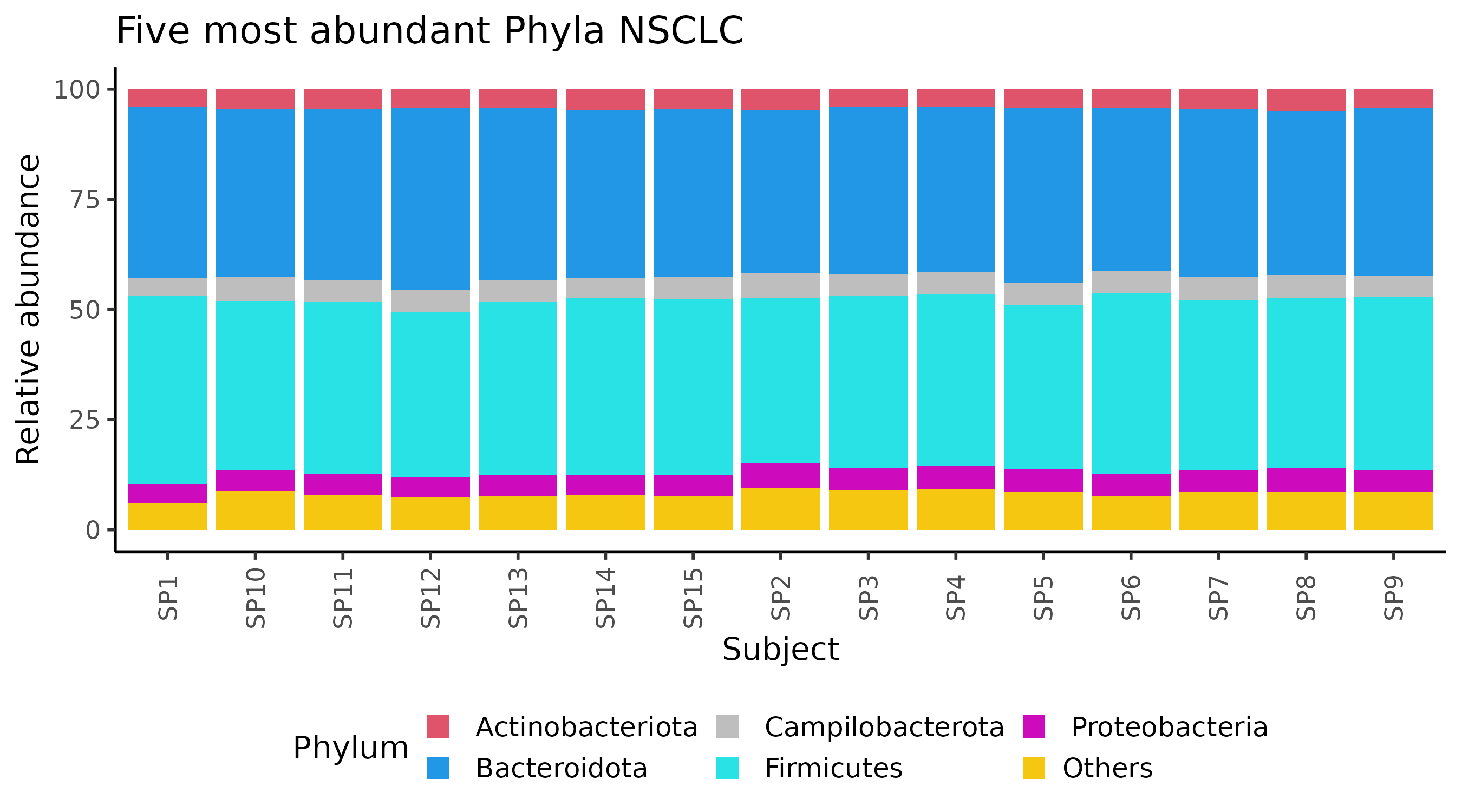

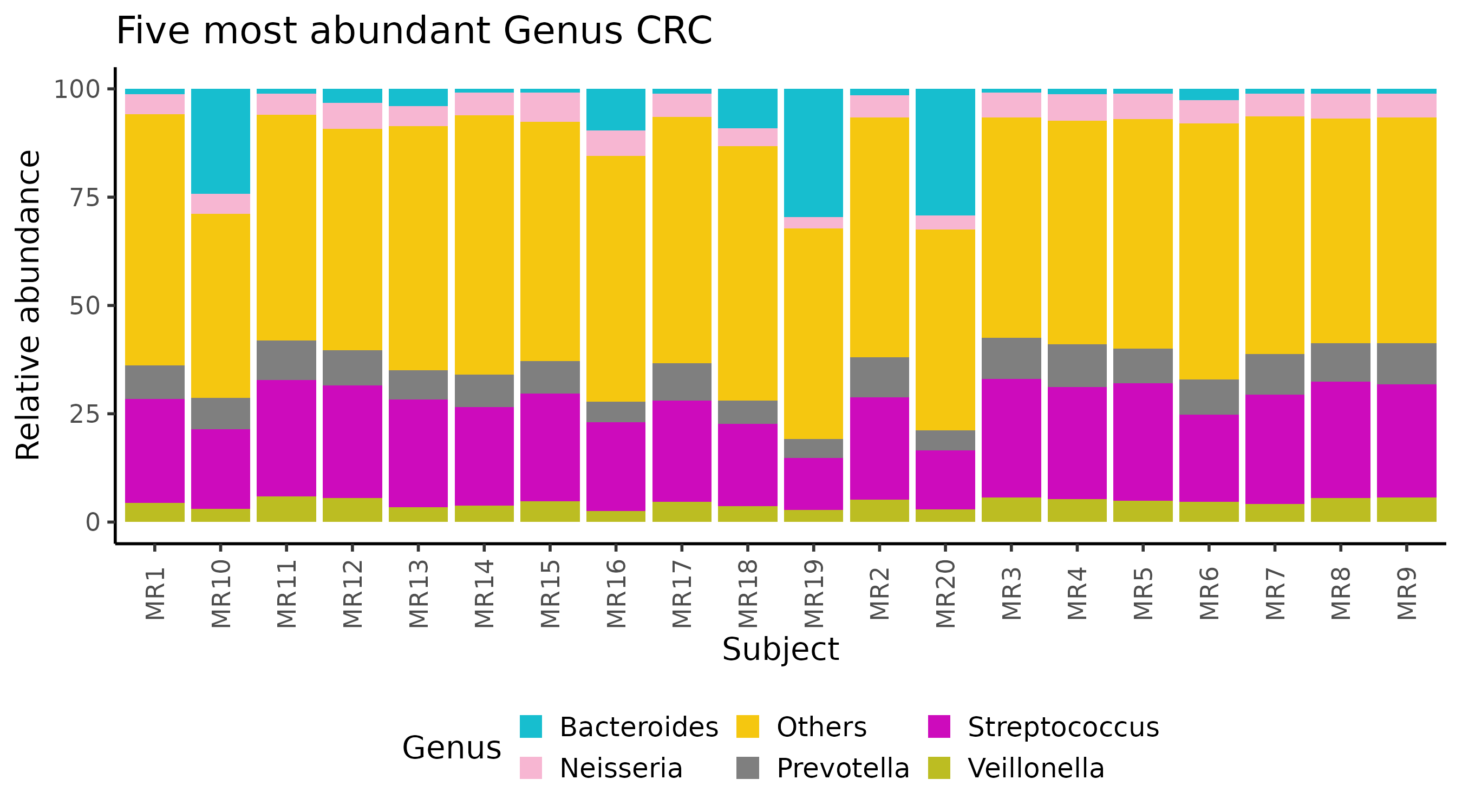

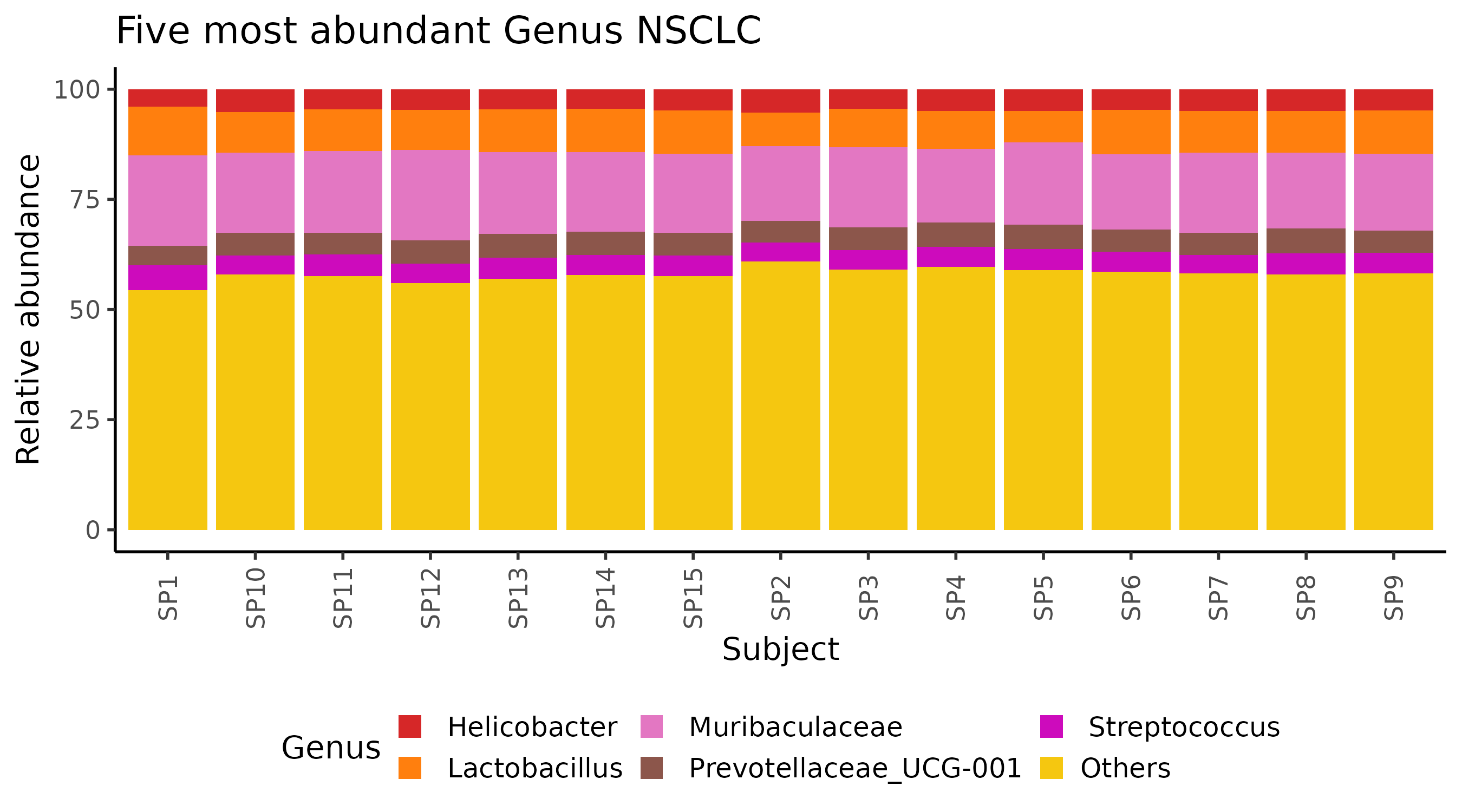

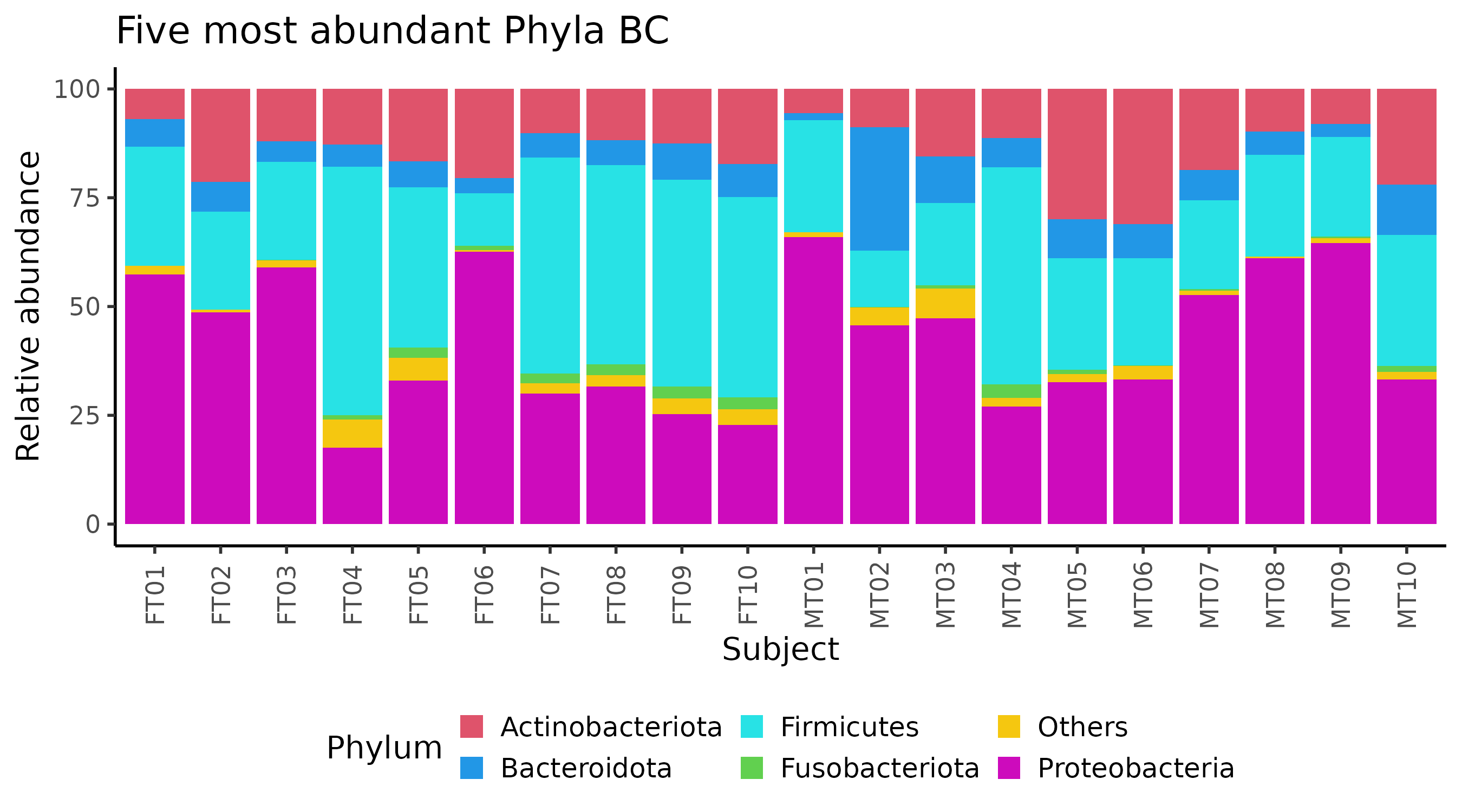


B)

E)

C)

F)

D)

**Figure S1**. Stacked bar plots displaying the average relative abundance of the top five phyla in BC (**A**), CRC (**B**) and NSCLC (**C**) samples and the of the top five genera in BC (**D**), CRC (**E**) and NSCLC (**F**) samples. BC: breast cancer, CRC: colorectal cancer, NSCLC: non-small cell lung cancer.

**
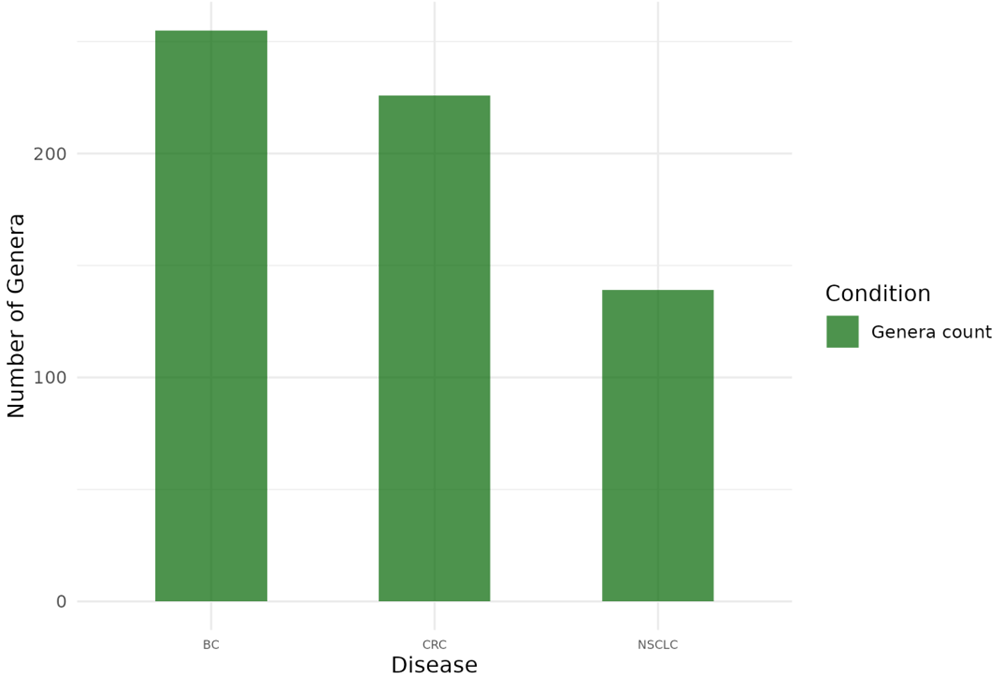
**

**Figure S2**. Histogram showing the richness of bacterial genera across the three tumor types. BC: breast cancer, CRC: colorectal cancer, NSCLC: non-small cell lung cancer.


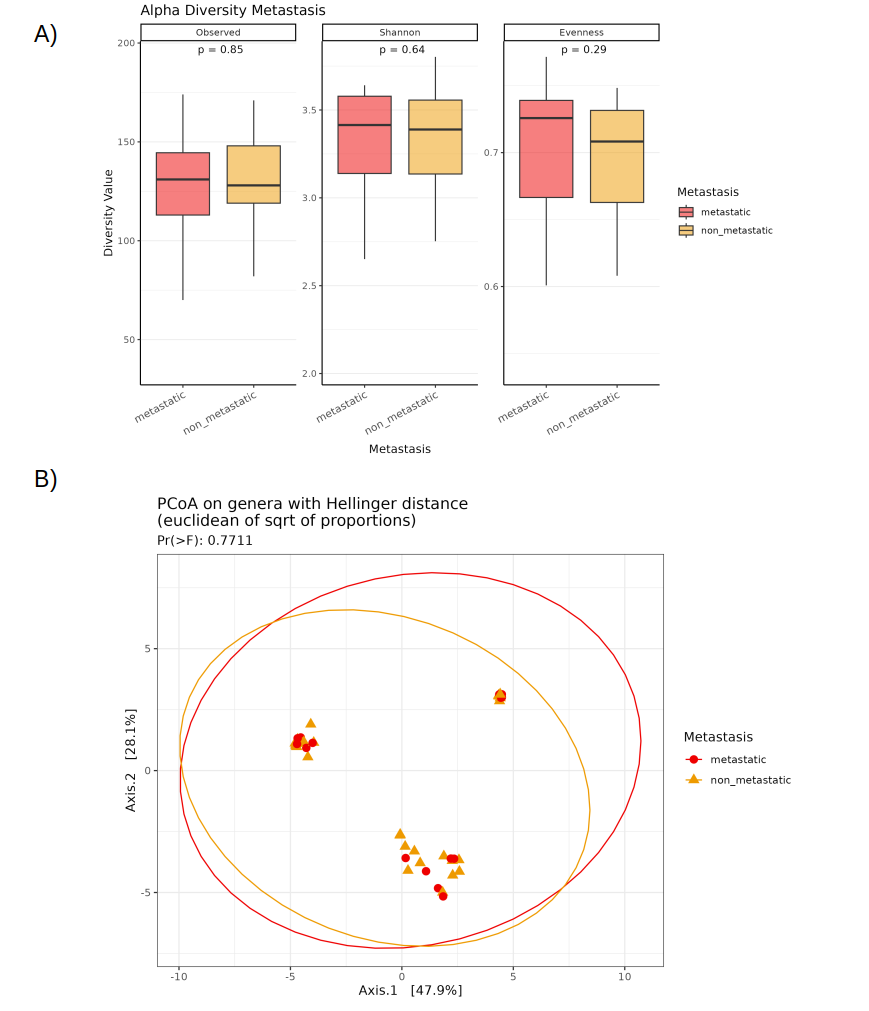


**Figure S3.** (**A**) Box plots illustrating alpha diversity indices (Observed ASV richness, Shannon index, and Pielou’s Evenness) in metastatic and non-metastatic groups. Statistical differences between groups were assessed using the Wilcoxon test, with p-values < 0.05 considered statistically significant. (**B**) Principal coordinate analysis (PCoA) conducted with the Hellinger distance on transformed genera abundances of metastatic and non metastatic samples.

**
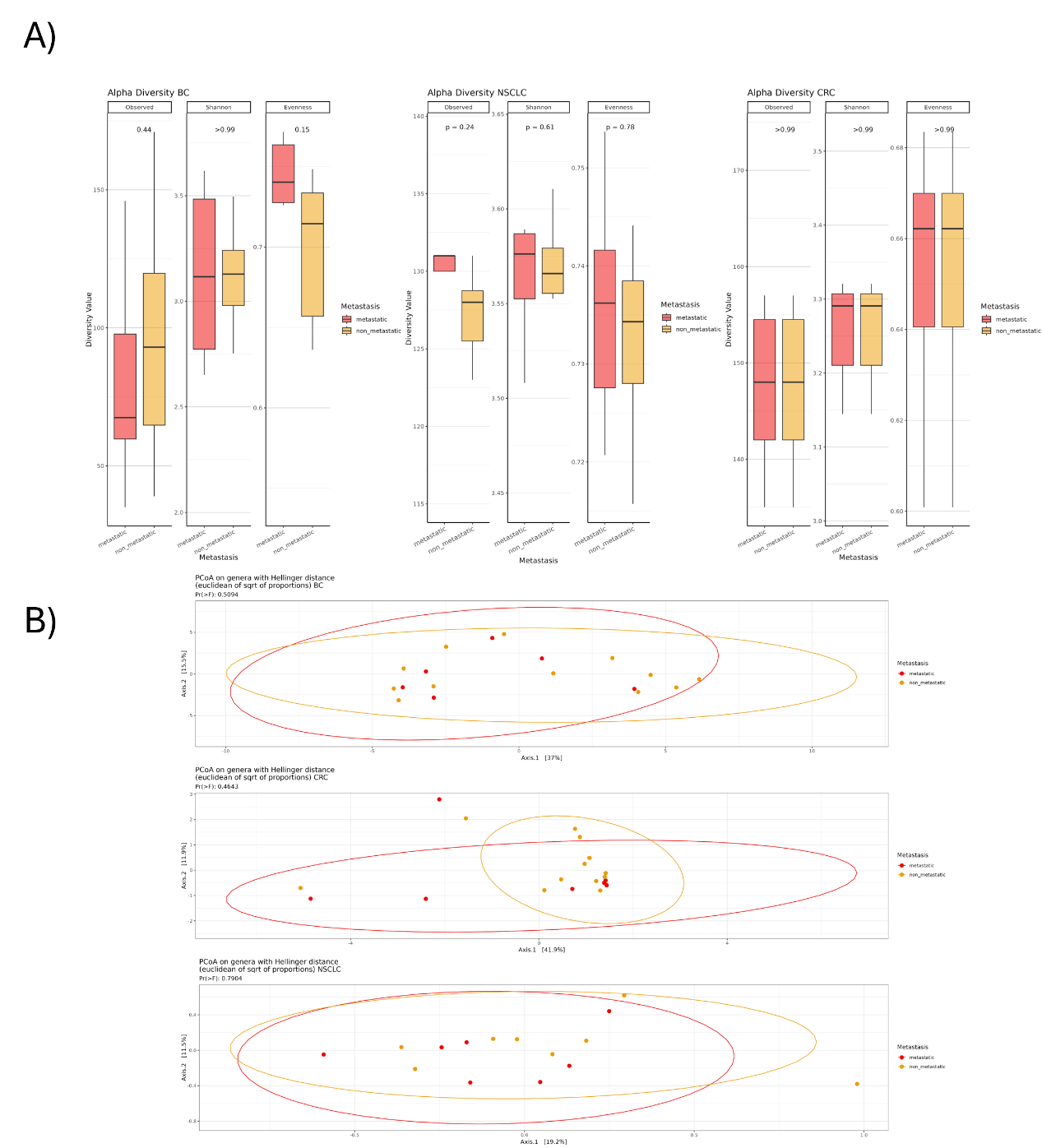
**

**Figure S4.** (**A**) Box plots illustrating alpha diversity indices (Observed ASV richness, Shannon index, and Pielou’s Evenness) between BC, CRC, and NSCLC samples, stratified into metastatic and non-metastatic groups. Statistical differences between groups were assessed using the Wilcoxon test, with p-values < 0.05 considered statistically significant. (**B**) Principal coordinate analysis (PCoA) conducted with the Hellinger distance on transformed genera abundances of BC, CRC and NSCLC samples. BC: breast cancer, CRC: colorectal cancer, NSCLC: non-small cell lung cancer.


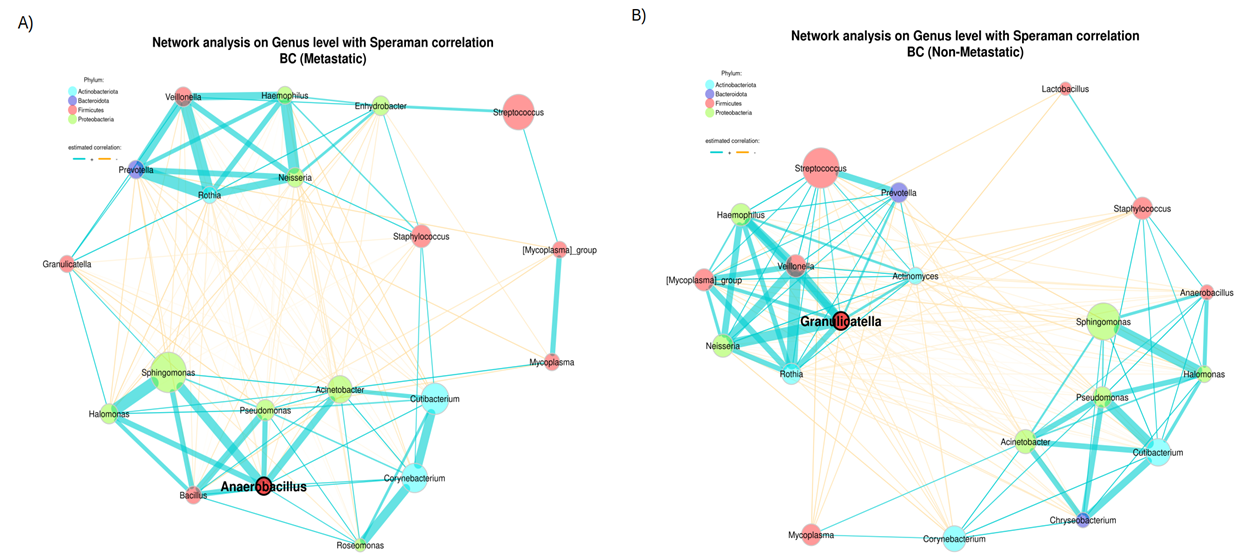


**Figure S5.** Interaction networks of the top 20 bacterial genera in the metastatic (**A**) and non-metastatic (**B**) BC groups. Nodes represent bacterial genera and are colored according to their respective phylum (see legend). Node size reflects the number of associations (degree centrality), with larger nodes indicating taxa with more interactions. The hub taxon—defined as the genus with the highest eigenvector centrality—is outlined in black. Edges represent significant associations between taxa, with **blue lines indicating positive correlations** and **orange lines indicating negative correlations**. The **thickness of the edges** corresponds to the strength of the correlation. The networks were constructed using centered log-ratio (CLR) normalized abundance data and visualized using a spring layout. BC: breast cancer.

**
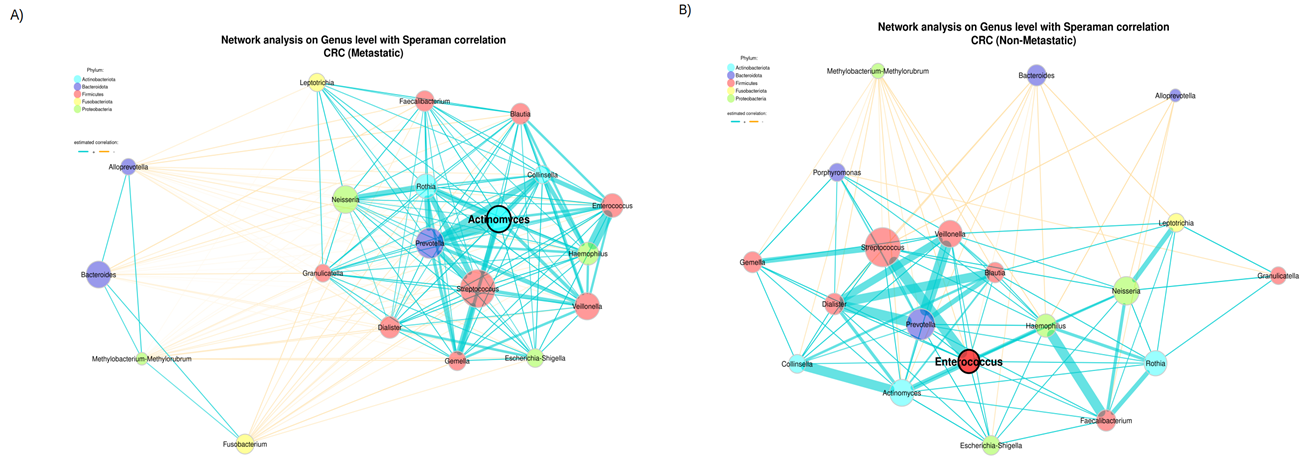
**

**Figure S6.** Interaction networks of the top 20 bacterial genera in the metastatic (**A**) and non-metastatic (**B**) CRC groups. Nodes represent bacterial genera and are colored according to their respective phylum (see legend). Node size reflects the number of associations (degree centrality), with larger nodes indicating taxa with more interactions. The hub taxon—defined as the genus with the highest eigenvector centrality—is outlined in black. Edges represent significant associations between taxa, with **blue lines indicating positive correlations** and **orange lines indicating negative correlations**. The **thickness of the edges** corresponds to the strength of the correlation. The networks were constructed using centered log-ratio (CLR) normalized abundance data and visualized using a spring layout. CRC: colorectal cancer.


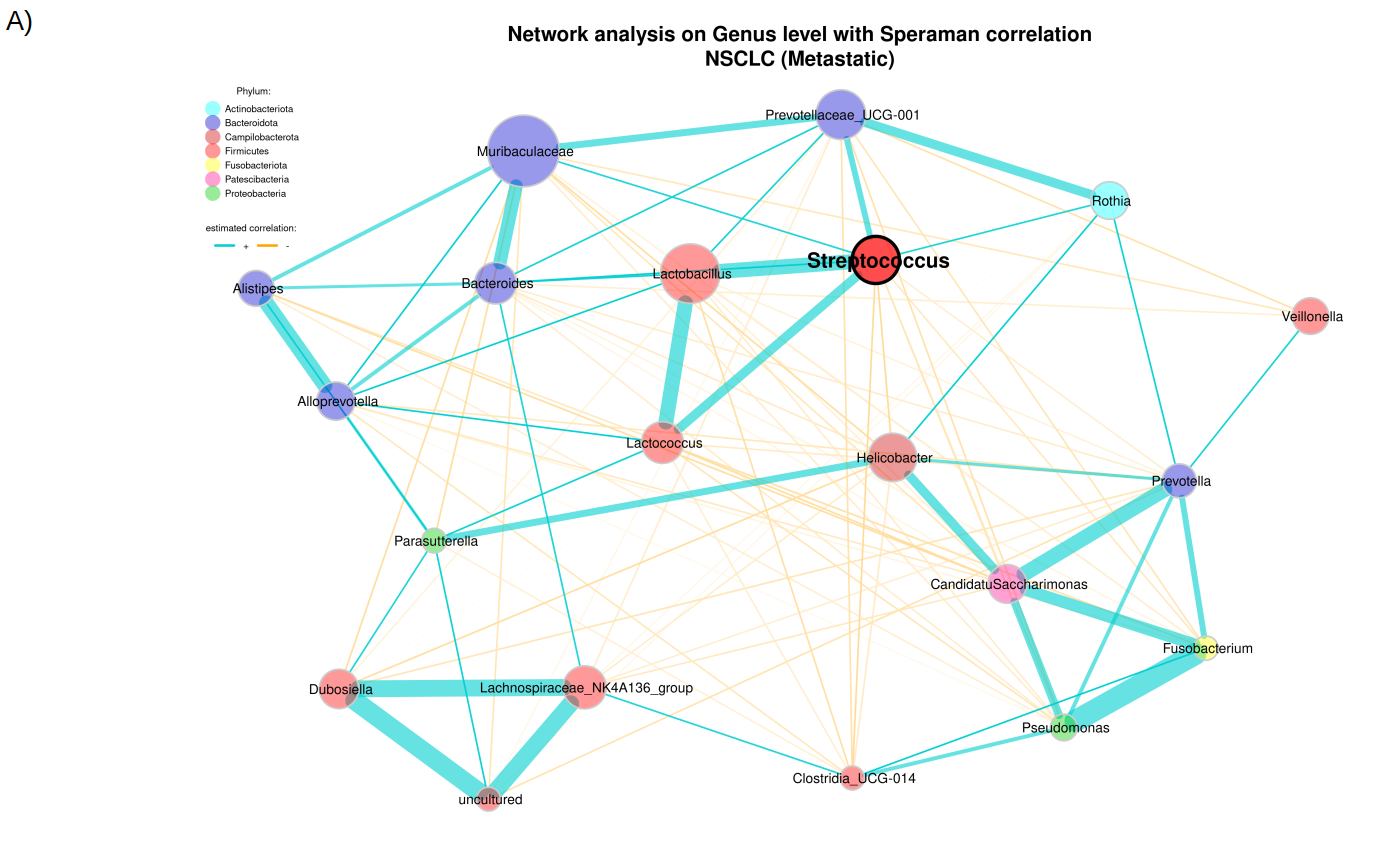

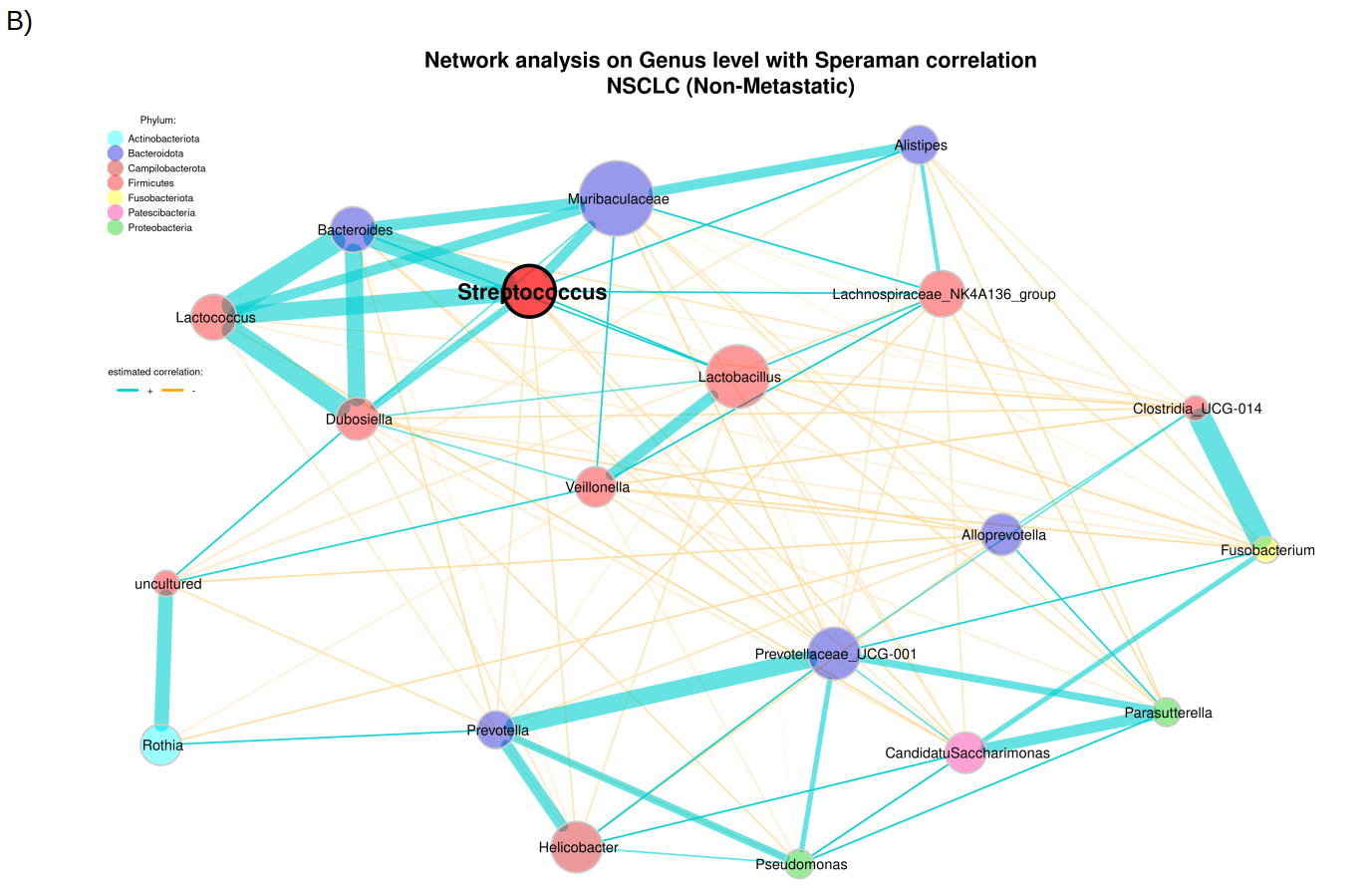


**Figure S7.** Interaction networks of the top 20 bacterial genera in the metastatic (**A**) and non-metastatic (**B**) NSCLC groups. Nodes represent bacterial genera and are colored according to their respective phylum (see legend). Node size reflects the number of associations (degree centrality), with larger nodes indicating taxa with more interactions. The hub taxon—defined as the genus with the highest eigenvector centrality—is outlined in black. Edges represent significant associations between taxa, with **blue lines indicating positive correlations** and **orange lines indicating negative correlations**. The **thickness of the edges** corresponds to the strength of the correlation. The networks were constructed using centered log-ratio (CLR) normalized abundance data and visualized using a spring layout. NSCLC: non-small cell lung cancer.

**SUPPLEMENTARY TABLES**

**Table S1.** Demographic and clinical characteristics of breast cancer patients.

| **Patient ID** | **Age** | **Gender** | **Stage/Metastasis** |
| --- | --- | --- | --- |
| BC 1 | 70 | F | pN0 |
| BC 2 | 64 | F | pN3a |
| BC 3 | 33 | F | pN1a |
| BC 4 | 47 | F | pN0 |
| BC 5 | 42 | F | pN0 |
| BC 6 | 46 | F | pN0 |
| BC 7 | 48 | F | pN0 |
| BC 8 | 42 | F | pN1a |
| BC 9 | 39 | F | pN0 |
| BC 10 | 79 | F | pN0 |
| BC 11 | 77 | M | pN0 |
| BC 12 | 48 | M | pNx |
| BC 13 | 57 | M | rpN0 |
| BC 14 | 48 | M | rpN0 |
| BC 15 | 69 | M | pN0 |
| BC 16 | 68 | M | pN2a |
| BC 17 | 76 | M | pN0 |
| BC 18 | 84 | M | pN2a |
| BC 19 | 88 | M | pNx |
| BC 20 | 82 | M | pN1a |

**Table S2.** Demographic and clinical characteristics of colorectal cancer patients.

| **Patient ID** | **Age** | **Gender** | **Stage/Metastasis** |
| --- | --- | --- | --- |
| MR 1 | 89 | F | pT4N0 |
| MR 10 | 83 | F | pT3N2b |
| MR 11 | 65 | M | pT3N2a |
| MR 12 | 59 | F | pT4N2a |
| MR 13 | 51 | M | pT3N0 |
| MR 14 | 54 | M | pT2N0 |
| MR 15 | 58 | F | pT3N0 |
| MR 16 | 58 | F | pT1N0 |
| MR 17 | 47 | F | pT3N0 |
| MR 18 | 48 | F | pT4N2b |
| MR 19 | 82 | M | pT4aN0 |
| MR 2 | 53 | F | pT3N0 |
| MR 20 | 81 | F | pT4aN2b |
| MR 3 | 57 | F | pT3N0 |
| MR 4 | 44 | F | pT3N0 |
| MR 5 | 50 | F | pT4N0 |
| MR 6 | 80 | F | pT2N0 |
| MR 7 | 49 | M | pT2N0 |
| MR 8 | 63 | M | pT3N2a |
| MR 9 | 74 | M | pT4aN2a |

**Table S3**. Demographic and clinical characteristics of non-small cell lung cancer patients.

| **Patient ID** | **Age** | **Gender** | **Stage/Metastasis** |
| --- | --- | --- | --- |
| SP 1 | 89 | M | No |
| SP 2 | 89 | M | No |
| SP 3 | 60 | M | No |
| SP 4 | 77 | F | Yes |
| SP 5 | 79 | M | Yes |
| SP 6 | 82 | F | No |
| SP 7 | 87 | F | No |
| SP 8 | 71 | M | Yes |
| SP 9 | 59 | F | Yes |
| SP 10 | 67 | F | No |
| SP 11 | 83 | M | No |
| SP 12 | 70 | F | Yes |
| SP 13 | 91 | M | Yes |
| SP 14 | 64 | M | Yes |
| SP 15 | 87 | M | No |
